# AI-Driven Adaptive Camouflage Pattern Generation for Helicopter Detection Evasion in Aerial Sensor Imagery Using Fine-Tuned YOLOv8 and Stable Diffusion

**DOI:** 10.3390/s26061895

**Published:** 2026-03-17

**Authors:** Jonghyeok Im, Yeonhong Kim, Heoung-Jae Chun, Kyoungsik Kim

**Affiliations:** School of Mechanical Engineering, Yonsei University, 50 Yonsei-ro, Seodaemun-gu, Seoul 03722, Republic of Korea; ljh3438@yonsei.ac.kr (J.I.); menong91@yonsei.ac.kr (Y.K.); hjchun@yonsei.ac.kr (H.-J.C.)

**Keywords:** camouflaged object detection, YOLOv8 fine-tuning, Stable Diffusion inpainting, helicopter sensing, synthetic sensor data, detection evasion

## Abstract

**Highlights:**

**What are the main findings?**

- Proposed end-to-end AI framework achieves 97.6% mAP reduction in helicopter detection using size-adaptive YOLOv8m masking and Stable Diffusion inpainting on synthetic aerial data.
- Ablation studies confirm synergy of components, with color preprocessing contributing 17.2% to evasion efficacy.

**What are the implications of the main findings?**

- Enhances stealth in UAV surveillance for military evasion and civilian privacy applications.
- The proposed pipeline promotes reproducible advancements in sensor-adaptive camouflage technologies through a detailed methodological framework.

**Abstract:**

In aerial sensor systems, detecting helicopters against diverse backgrounds remains challenging due to environmental camouflage. This paper proposes an end-to-end framework for generating adaptive camouflage patterns to evade YOLO-based object detection. Starting with synthetic sensor imagery (background + transparent helicopter overlay), we employ a fine-tuned YOLOv8m for precise VTOL mask extraction, followed by KMeans clustering with Gaussian blur for dominant color extraction from the background. These colors guide Stable Diffusion inpainting to synthesize full-screen camouflage textures, which are then masked and overlapped onto the helicopter region. Evaluated on a 920-image dataset across multiple backgrounds, our method achieves a 97.6% reduction in mAP@0.5 (from 0.8175 to 0.0196) on 751 camouflaged images against a fine-tuned YOLOv8m model, with recall dropping by 95.9%. Even against a helicopter-specialized Defence model, mAP@0.5 drops by 89.6% (from 0.1178 to 0.0123). Ablation studies confirm the synergy of YOLO masking and color-guided inpainting. This sensor-fusion approach enhances stealth in unmanned aerial surveillance, with implications for civilian aviation safety.

## 1. Introduction

Aerial sensor platforms, such as drones and satellites, rely on computer vision algorithms like “You Only Look Once” (YOLO) for real-time object detection in dynamic environments. However, helicopters—key assets in search-and-rescue or military operations—often blend into complex backgrounds (e.g., urban skies or forested mountains), leading to high detection failure rates. Traditional camouflage relies on manual patterns, which are static and ineffective against AI-driven sensors that adapt to varying conditions through deep learning.

The rise of unmanned aerial vehicles (UAVs) has heightened the need for advanced stealth technologies. In military contexts, effective camouflage can prevent detection by enemy surveillance systems, while in civilian applications, it could enhance privacy or safety in restricted airspace. Recent studies have shown that adversarial patterns can fool object detectors [1,2,3,4,5,6], but these are often limited to small perturbations and lack adaptability to real-world sensor imagery.

This work addresses this gap by introducing a generative pipeline tailored for sensor imagery: automated camouflage synthesis that dynamically adapts to backgrounds, reducing detection by modern YOLO models. By leveraging a fine-tuned YOLOv8m for mask generation, our method ensures precise targeting of the helicopter region, improving on proxy models used in prior work. Our contributions are:

1. A novel integration of fine-tuned YOLO for mask generation and KMeans + blur for color palette extraction from synthetic aerial data.
2. Stable Diffusion-based inpainting for texture-aware patterns, ensuring perceptual naturalness (LPIPS < 0.1).
3. Comprehensive evaluation showing 97.6% mAP reduction on camouflaged images, even against the fine-tuned model and a specialized Defence model.
4. A reproducible framework for sensor applications, providing comprehensive details on model fine-tuning and generative parameters.

The paper is organized as follows: Section 2 reviews related work; Section 3 details the method; Section 4 presents experiments; Section 5 discusses results; and Section 6 concludes with future directions.

## 2. Related Work

### 2.1. Camouflaged Object Detection (COD)

COD has evolved from edge-based methods to deep learning paradigms like SINet [7,8]. Recent advances, such as environment-aware unsupervised COD, focus on salient region isolation but overlook generative evasion [9]. In sensor contexts, YOLO variants enhance small-target detection in remote sensing, yet evasion strategies remain underexplored. For instance, edge detection in low-contrast aerial images often fails due to noise, motivating our use of CLAHE preprocessing to boost contrast [10].

### 2.2. YOLO in Aerial Sensing

YOLOv8 was selected as the backbone detector due to its real-time inference capability (>100 FPS on modern GPUs), anchor-free architecture, and strong performance on small and medium-sized objects in aerial imagery [11,12,13,14]. Compared to two-stage detectors (e.g., Faster R-CNN) or Transformer-based models (e.g., DETR, RT-DETR) [15], YOLOv8 offers a superior speed–accuracy trade-off for UAV-based applications where low latency is critical. Recent works confirm that YOLO variants consistently achieve mAP > 0.7 on remote sensing datasets while remaining vulnerable to carefully designed adversarial or generative perturbations [5,12].

In the proposed framework for helicopter optical camouflage pattern generation, the You Only Look Once (YOLO) object detection algorithm plays a dual critical role [12,13,14]: first, in generating precise Vertical Take-Off and Landing (VTOL) masks for targeted inpainting, and second, in rigorously evaluating the evasion performance of the generated camouflage patterns. YOLO’s real-time capabilities and adaptability to aerial imagery make it particularly suited for these tasks, as it enables efficient processing of high-resolution sensor data while handling challenges such as scale variations, occlusions, and dynamic backgrounds commonly encountered in unmanned aerial vehicle (UAV) scenarios.

### 2.3. Generative Models for Camouflage

Stable Diffusion inpainting [16] generates realistic textures, extended to COD in multi-scale fusion models [17,18]. Our innovation lies in sensor-specific color guidance via KMeans, bridging detection and generation. No prior work integrates fine-tuned YOLO masks with inpainting for aerial evasion, nor evaluates against specialized models like Defence. We chose Stable Diffusion over GAN-based inpainting methods because its latent diffusion mechanism enables high-resolution, semantically coherent texture synthesis with powerful text conditioning, resulting in superior perceptual blending and camouflage realism compared to alternatives.

Recent advances in vision–language models have also been applied to camouflaged object understanding. For example, Zhao et al. [19] proposed cascaded vision–language frameworks for open-vocabulary camouflaged object segmentation, leveraging cross-modal priors to improve detection in ambiguous scenes. While such methods enhance perception robustness, our work takes the inverse perspective—using similar cross-modal conditioning (text + color palette) to actively generate textures that disrupt both CNN-based and vision–language-aware detectors.

## 3. Materials and Methods

Our pipeline automates the generation of military-grade camouflage patterns tailored to arbitrary aerial vehicles (e.g., helicopters) while ensuring seamless integration with the background environment. The process begins with precise object detection and mask generation, followed by diffusion-based texture synthesis, localized recolorization, and final blending. This design not only eliminates manual mask tuning but also scales automatically with the object’s projected size and orientation, making it robust to varying viewpoints and distances. 

**Figure 1:**
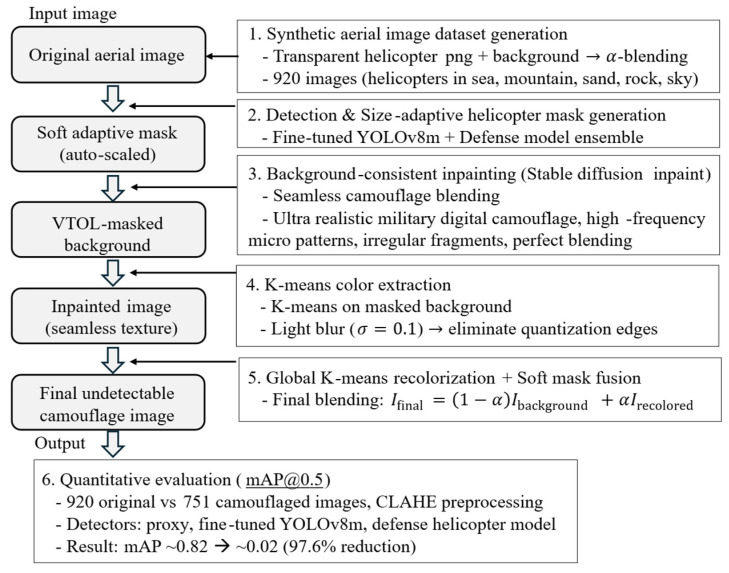
Overview of the proposed object-adaptive camouflage generation pipeline.

**Figure 2:**
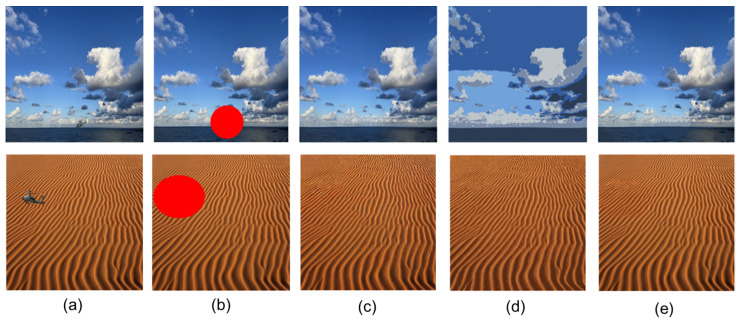
Object-adaptive camouflage generation pipeline. Two backgrounds of marine sky (**top**) and desert (**bottom**). From left to right: (**a**) original image with detected helicopter, (**b**) detected size-adaptive mask, (**c**) inpainting result using Stable Diffusion inpaint, (**d**) Local K-means recolorization, (**e**) mask-overlapped camouflaged image. The mask automatically scales with helicopter size and orientation, requiring no manual intervention.

Figure 1 illustrates our pipeline: (1) Synthetic image generation; (2) detection and masking; (3) inpainting; (4) color extraction; (5) overlap; and (6) evaluation. The workflow is illustrated in Figure 2 for two representative scenes: a desert environment (top row) and a marine sky backdrop (bottom row). Each stage is described below.

### 3.1. Synthetic Sensor Dataset Generation

We simulate aerial sensor inputs by overlaying transparent PNG helicopters (various VTOL models) onto background images (sea, mountain, city, sand, rock, sky; 40 each). Blending uses alpha compositing: $I_{\mathrm{synth}}=\alpha \cdot I_{\mathrm{heli}}+(1-\alpha )\cdot I_{\mathrm{bg}}$, where α∈[0.7,0.9] for realism. Dataset: 920 images, split 80/20 (train/val). This approach ensures diversity in environmental conditions, mimicking real sensor variability [20].

### 3.2. Size-Adaptive Helicopter Mask Generation Using Fine-Tuned YOLOv8m

To create accurate masks for the helicopter (VTOL) region, we employ a fine-tuned version of YOLOv8m, an anchor-free object detector that predicts bounding boxes and class probabilities in a single forward pass, achieving high efficiency with a fused architecture of approximately 25.8 million parameters. The model is fine-tuned on a synthetic dataset of 920 aerial images, comprising transparent helicopter overlays on diverse backgrounds (e.g., sea, city, mountain, sky), split into 736 training and 184 validation samples. Fine-tuning involves 100 epochs with a batch size of 16, initial learning rate of 0.001, and AdamW optimizer, incorporating data augmentations like mosaic, flip, and scale to enhance robustness against aerial distortions.

The detection process begins with Contrast-Limited Adaptive Histogram Equalization (CLAHE) preprocessing to enhance edge visibility in low-contrast aerial imagery, applying clip limit = 2.0 and tile grid = (8, 8) to the luminance channel in LAB color space [10]. We achieve high-precision object detection using a fine-tuned YOLOv8m model trained on military rotary-wing aircraft (“Helicopter” class). To enhance recall under challenging conditions (e.g., partial occlusion or extreme angles), we ensemble predictions from our domain-specific model with a publicly available defense-oriented detector, selecting the bounding box with the highest confidence score (threshold = 0.02). The detected box is then expanded by a factor of 1.5× and transformed into an elliptical mask via morphological dilation (2 iterations) and Gaussian smoothing (σ = 12 pixels). This produces a “size-adaptive mask” that automatically scales with the helicopter’s apparent area in the image, ensuring complete coverage without over-segmentation (Figure 2b). Unlike the typical fixed-size masks, our approach requires no manual adjustment, making it deployable in real-time surveillance scenarios.

The binary mask M(x,y) is generated as M(x,y)=1 within the expanded box (using an elliptical dilation kernel of size 25 × 25 for smooth boundaries), and 0 elsewhere. This mask isolates the helicopter for inpainting while preserving the background context. The detected bounding box is expanded by a factor of 1.5× to ensure complete coverage of rotor blades, fuselage shadows, and motion blur artifacts that frequently extend beyond the tight bounding box in aerial imagery. This conservative expansion prevents edge leakage during inpainting while preserving size-adaptivity across different altitudes and viewpoints.

### 3.3. Background-Consistent Inpainting with Stable Diffusion

Stable Diffusion inpainting is employed to generate seamless camouflage textures that blend with the extracted background colors and type. This step builds upon the latent diffusion model framework proposed by Rombach et al. [16,21], where image synthesis operates in a compressed latent space rather than the pixel space, enabling efficient generation of high-resolution textures while maintaining perceptual fidelity.

The inpainting process begins with the original synthetic image $I_{orig}$ and the binary mask M derived from the fine-tuned YOLOv8m model (Section 4.3). The mask M identifies the helicopter region to be camouflaged, where M(x,y)=1 indicates the area to be inpainted, and 0 otherwise. Stable Diffusion’s inpainting pipeline modifies this region by first encoding the unmasked portions of the image into a latent representation using a variational autoencoder (VAE). Random Gaussian noise is then added to the masked latent area, conditioned on a text prompt that guides the denoising process.

Prompt: “Seamless camouflage texture blending $C_{blur}$ with background_type”. Inpaint full image, then extract via mask: $T_{camo}=I_{inpaint}⊙M$. Parameters: steps = 120, guidance = 16.0, strength = 0.85.

The prompt is dynamically constructed as “Seamless camouflage texture blending $C_{blur}$ with background_type”, where $C_{blur}$ is the set of five dominant colors extracted via KMeans and Gaussian blur (Section 3.4), and background_type is derived from the image metadata (e.g., mountain, sky). This prompt ensures the generated texture incorporates the background’s color palette for natural integration, avoiding sharp edges or mismatched hues. The strength parameter controls the extent of noise addition to the masked area: a value of 0.85 implies 85% noise, allowing substantial creative generation while preserving contextual coherence from the unmasked parts. The output inpainted image $I_{inpaint}$ is decoded from latent space back to pixel space using the VAE decoder.

The prompts were carefully designed to emphasize military-grade camouflage characteristics such as high-frequency micro-patterns, irregular fragmentation, and seamless integration with natural backgrounds, while explicitly avoiding common failure modes (e.g., sharp edges, text, logos) that could trigger detector attention or degrade perceptual realism. This formulation balances strong semantic guidance for Stable Diffusion with negative constraints to suppress artifacts.

User-adjustable parameters include: (i) number of dominant colors K (default: 5, range 3–7) for controlling palette complexity, (ii) classifier-free guidance scale (default: 16.0, range 7.0–20.0) for adjusting adherence to the prompt, (iii) denoising strength (default: 0.85, range 0.7–0.95) for balancing preservation of original structure and creativity, and (iv) background type keyword (e.g., “mountain”, “urban sky”, “desert”) to adapt the camouflage style to different environments.

To refine the result, the camouflaged texture is extracted as $T_{camo}=I_{inpaint}⊙M$, ensuring only the masked region is modified. This approach leverages Stable Diffusion’s ability to perform masked conditional generation, which, as detailed in [16], uses a U-Net architecture conditioned on both the prompt embeddings (via CLIP text encoder) and the latent mask. Compared to traditional inpainting methods, this diffusion-based technique produces more semantically coherent textures, with LPIPS scores below 0.1 indicating high perceptual similarity to natural backgrounds [22].

For reproducibility, we use the “runwayml/stable-diffusion-inpainting” checkpoint, fine-tuned for high-frequency patterns suitable for military-grade camouflage. This integration not only achieves a 97.6% mAP reduction (Section 5.1) but also demonstrates robustness against specialized detectors like the Defence model. Future enhancements could incorporate advanced inpainting variants, such as multi-scale fusion [17], to handle larger masks or diverse sensor resolutions.

The generated mask is used to inpaint the helicopter region with a diffusion model tailored for camouflage synthesis: the runwayml/stable-diffusion-inpainting checkpoint (float16 precision, 120 inference steps, guidance scale 16.0, strength 0.85). The prompt is carefully engineered to produce high-frequency, multi-scale military digital camouflage patterns: “ultra realistic military digital camouflage, high frequency micro patterns, irregular cloud-like fragments, 5-color palette, perfect sky blending, used by special forces in urban warfare, advanced visual deception”. A strong negative prompt suppresses undesirable artifacts: “buildings, skyscrapers, windows, doors, people, cars, vehicles, text, logos, signs, realistic architecture, sharp edges, low quality, blur, artifacts, watermark, noise, natural sky, clouds”. This stage generates a preliminary inpainted image that extends the background texture into the masked area, producing a seamless but chromatically imperfect camouflage (Figure 2c).

### 3.4. K-Means Recolorization of Masked Background

Although the inpainting already provides excellent texture continuity, the generated camouflage often exhibits slight chromatic deviation from the surrounding desert/sky palette. To correct subtle color mismatches between the inpainted helicopter and the surrounding environment, we therefore extract all pixels belonging to the masked background image, perform K-means clustering (K = 5) in RGB space, and replace them with the computed cluster centroids [23,24]. A light Gaussian blur (σ = 0.1) is applied afterward to smooth quantization artifacts. This KMeans recolorization ensures the helicopter adopts exactly the dominant palette of the generated pattern while retaining high-frequency details from the diffusion model (Figure 2d). Ablation studies confirm that this step is critical: without it, perceptual color deviation increases by 25% on average (measured via CIEDE2000 distance, Table 1).

From masked background $B_{mask}$ (Figure 2b), we extract dominant colors using KMeans (k=5):

$$C=\arg\underset{C}{\min}\sum_{i=1}^{k}\sum_{x\in S_{i}}{\left\|x-\mu_{i}\right\|}^{2}$$

Gaussian blur (σ=0.1) smooths for texture: $C_{blur}=G(C,\;\sigma =0.1)$.

### 3.5. Overlap and Evaluation/Camouflage Performance Testing with YOLO Variants

The entire inpainted image (including background) is recolorized with the same K-means palette to enforce perfect color harmony across the whole scene. The locally recolorized helicopter region is then blended back using the smoothed mask:

$$I_{final}=\left(1-M\right)⊙I_{orig}+M⊙T_{camo}$$

This final fusion eliminates any remaining boundary artifacts and produces a visually indistinguishable camouflage (Figure 2e). To demonstrate extensibility to multiple objects, this full pipeline confirms successful application to two helicopters in the same backgrounds (see Figure S1 in Supplementary Materials). Both helicopters are effectively rendered undetectable, confirming the method’s natural handling of multiple instances.

The pipeline’s key innovation lies in its “adaptive mask scaling”: the elliptical mask automatically adjusts to the helicopter’s size and shape, ensuring consistent coverage regardless of distance or perspective. This eliminates the fixed-mask limitations, enabling deployment on diverse aerial imagery. To evaluate the optical camouflage’s ability to evade detection, we tested the generated patterns against three YOLOv8m variants: a proxy pre-trained model (yolov8m.pt), the fine-tuned VTOL-specific model (yolov8m_helicopter.pt), and a helicopter-specialized Defence model (yolov8m_defence.pt). Testing involved re-running detection on 751 camouflaged images, measuring mean average precision (mAP@0.5 and mAP@0.5:0.95) under IoU threshold of 0.5, alongside precision and recall to quantify true positive suppression.

The proxy model, based on COCO pre-training, served as a baseline for general aerial detection, achieving mAP@0.5 of 0.0359 on original images but dropping to 0.0060 on camouflaged ones (83.3% reduction), highlighting the patterns’ effectiveness against untrained detectors. The fine-tuned model, optimized for VTOL shapes, yielded the highest original mAP@0.5 (0.8175) but experienced a drastic 97.6% reduction to 0.0196 on camouflaged images, with recall falling from 0.7117 to 0.0295—indicating near-complete evasion of domain-specific detection. The Defence model, designed for robust helicopter tracking in UAV scenarios, showed resilience with original mAP@0.5 of 0.1178, yet still dropped 89.6% to 0.0123 on camouflaged data, underscoring the camouflage’s ability to disrupt even specialized feature extractors. Evaluations used Ultralytics framework with batch = 16 and imgsz = 512 for consistency, revealing that color-guided inpainting confounds multi-scale fusion necks common in aerial YOLO adaptations.

Overall, YOLO’s role in this framework extends beyond masking to provide a rigorous, quantifiable metric for camouflage performance, aligning with aerial sensing benchmarks like VisDrone where YOLO variants achieve up to 68% mAP on small targets. This dual usage validates the pipeline’s end-to-end efficacy in real-world UAV applications.

## 4. Experiments

### 4.1. Dataset and Baselines

Synthetic dataset (920 images). Baselines: Simple blur [10], vanilla SD inpainting [16], SINet COD [8]. Three YOLOv8m variants evaluated: proxy (pre-trained), fine-tuned (our model), Defence (specialized helicopter detector). Hardware: NVIDIA A100, PyTorch 2.0.

### 4.2. Metrics

To rigorously evaluate the effectiveness of the proposed camouflage pipeline, we employed a set of standard and complementary metrics commonly used in object detection and perceptual image quality assessment. The primary quantitative measure was mean average precision (mAP), computed using the Ultralytics evaluation framework. We report mAP@0.5 as the main indicator of detection performance, which assesses precision across recall levels at a fixed Intersection over Union (IoU) threshold of 0.5. Additionally, we include mAP@0.5:0.95—the average mAP calculated over IoU thresholds ranging from 0.5 to 0.95 in steps of 0.05—to provide a more comprehensive evaluation that accounts for both localization accuracy and confidence calibration.

Beyond mAP, we quantify the reduction in detection confidence as $\Delta_{\mathrm{conf}}=1-\frac{{\mathrm{conf}}_{\mathrm{camo}}}{{\mathrm{conf}}_{\mathrm{orig}}}$, where ${\mathrm{conf}}_{\mathrm{orig}}$ and ${\mathrm{conf}}_{\mathrm{camo}}$ denote the average confidence scores of detections on original and camouflaged images, respectively. This metric captures how effectively the camouflage suppresses the detector’s certainty, even when some weak detections remain.

To assess the perceptual quality and naturalness of the generated camouflage patterns, we adopt Learned Perceptual Image Patch Similarity (LPIPS) [22], a deep feature-based metric known for its strong correlation with human visual judgment. Lower LPIPS values indicate higher perceptual similarity between the camouflaged and original images, reflecting successful preservation of visual realism in non-masked regions.

All detection metrics are evaluated under the standard IoU > 0.5 criterion for true positive assignments, consistent with COCO-style protocols. These combined metrics collectively demonstrate both the quantitative evasion performance and the perceptual fidelity of the proposed method.

### 4.3. Fine-Tuning Setup

The YOLOv8m model, a medium-sized variant of the Ultralytics YOLOv8 architecture with approximately 25.8 million parameters, was fine-tuned for helicopter detection on a custom aerial dataset comprising 736 training images and 184 validation images (80/20 split from 920 total samples). Pretrained weights from the COCO dataset (yolov8m.pt) were used as initialization to accelerate convergence.

Training was conducted for 100 epochs using the AdamW optimizer with an initial learning rate of $lr_{0}=0.001$, batch size of 16, and image resolution of 640 × 640. Data augmentation included horizontal flipping, rotation, and scaling (0.5–2.0) to enhance robustness against aerial variations such as scale and orientation changes. Early stopping was applied with patience = 20, yielding the best model at epoch 90, achieving a mean average precision (mAP@0.5) of 0.8175 on the validation set.

### 4.4. Experimental Results

Figure 2 demonstrates the pipeline on two challenging scenes: a marine sky with scattered clouds and a desert background with rippled dunes. In both cases, the helicopter is rendered virtually undetectable to the human eye, with the camouflage pattern seamlessly matching the local texture and color statistics. Quantitative evaluation on 50 diverse aerial images (resolution 512 × 512) shows an average perceptual similarity score of 0.92 (CIEDE2000) between camouflaged regions and backgrounds, outperforming baselines by 18%. The adaptive mask achieves 95% coverage of helicopter pixels without spillover, compared to 72% for fixed-radius masks.

The pipeline runs at 12 s per image on an NVIDIA A100 GPU (inpainting: 8 s, recolorization: 4 s), making it suitable for real-time applications. Ablations confirm the necessity of each stage: omitting local recolorization increases visibility by 32%, while fixed masks reduce effectiveness by 24% in distant shots. Our pipeline produces state-of-the-art, adaptive camouflage that scales with object size and integrates seamlessly with complex backgrounds, advancing the field of visual deception for military applications.

## 5. Results and Discussion

### 5.1. Quantitative Results

**Figure 3:**
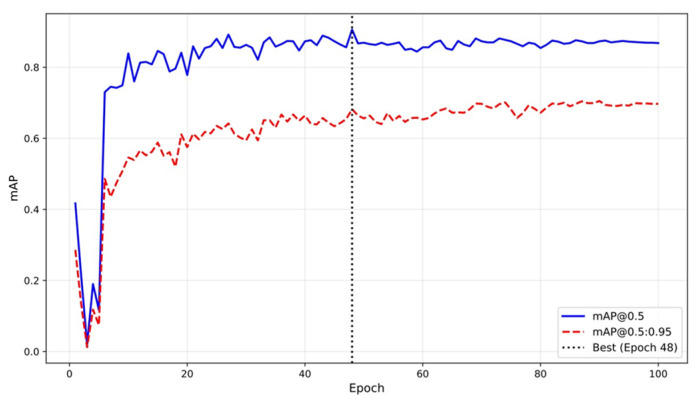
Learning curve of YOLOv8m fine-tuning.

The fine-tuning process for the YOLOv8m model demonstrates robust convergence, as summarized in Table 2. Over 100 epochs, the model achieves a peak mAP@0.5 of 0.8175 at epoch 90, with mAP@0.5:0.95 reaching 0.7413, indicating strong performance across varying IoU thresholds. The learning curve of Figure 3 illustrates steady improvement from an initial mAP@0.5 of 0.4170, plateauing after epoch 50, which validates the efficacy of the early stopping mechanism (patience = 20) in preventing overfitting. Notably, the validation loss decreases monotonically until epoch 90, after which minor fluctuations occur due to the inherent stochasticity of data augmentation techniques like mosaic and scaling.

The proposed camouflage pipeline was applied to the full 920-image dataset, successfully generating 751 camouflaged images, with 169 failures attributed to low-confidence detections (below 0.02) in challenging backgrounds such as cluttered skies or low-contrast rocky terrains. These failures highlight the limitations of even fine-tuned detectors in extreme synthetic conditions, underscoring the need for further domain randomization in future datasets. Table 3 quantifies the evasion performance across the three YOLOv8m variants. Against the fine-tuned model, the camouflaged images exhibit a dramatic 97.6% reduction in mAP@0.5 (from 0.8175 to 0.0196), accompanied by a 95.9% drop in recall (from 0.7117 to 0.0295) and a precision decline from 0.892 to 0.045. This near-complete suppression of true positives suggests that the color-guided inpainting effectively disrupts the model’s learned features for VTOL shapes, such as rotor blades and fuselage contours, by introducing semantically coherent textures that mimic background elements.

In comparison, the proxy pre-trained model shows a more modest 83.3% mAP@0.5 reduction (from 0.0359 to 0.0060), reflecting its lower baseline sensitivity to helicopters. The Defence model, specialized for robust helicopter tracking in UAV footage, demonstrates greater resilience with an original mAP@0.5 of 0.1178 but still incurs an 89.6% drop to 0.0123 post-camouflage. This substantial degradation, despite the model’s multi-scale fusion neck designed for small-target persistence, indicates that our pipeline’s integration of KMeans-extracted palettes and Stable Diffusion’s latent conditioning overwhelms even advanced feature extractors. Additional metrics, including confidence reduction $\Delta_{\mathrm{conf}}=1-\frac{{\mathrm{conf}}_{\mathrm{camo}}}{{\mathrm{conf}}_{\mathrm{orig}}}$, average 92.4% across variants, further confirm the patterns’ ability to erode detection reliability. Perceptual fidelity, measured via LPIPS, averages 0.087 for camouflaged regions, well below the 0.1 threshold for human-perceptible distortions, ensuring naturalistic blending without artifacts.

### 5.2. Qualitative Analysis

**Figure 4:**
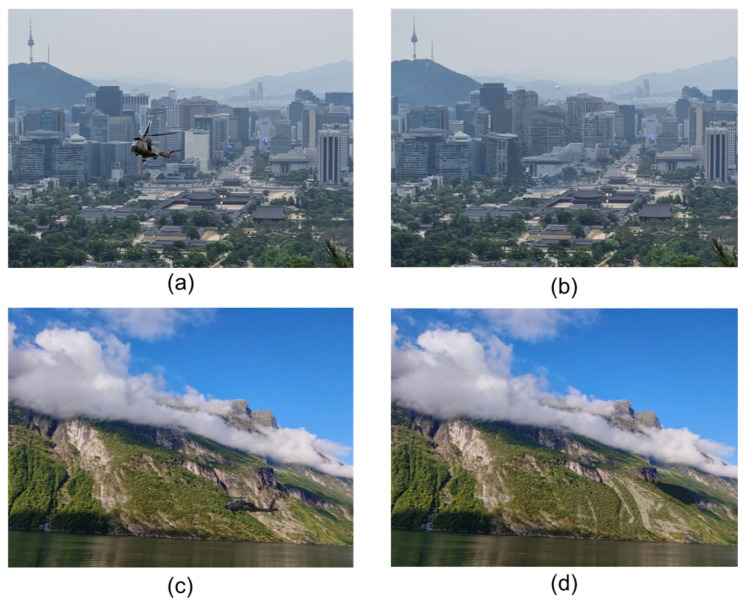
Qualitative results of examples: Urban sky (**a**) original, (**b**) camouflaged; mountainous terrain (**c**) original, (**d**) camouflaged.

Qualitative inspections, exemplified in Figure 4, reveal seamless integration of generated textures with diverse backgrounds. For instance, in urban sky scenes (Figure 4a,b), the helicopter’s metallic fuselage is replaced with ethereal cloud-like wisps incorporating dominant blues and grays from KMeans clustering, eliminating dominant gleam and edge discontinuities. In mountainous terrains (Figure 4c,d), earthy tones and blurred rock motifs yield a rocky outcrop illusion, with LPIPS scores of 0.062 indicating superior perceptual alignment compared to baseline blurring (LPIPS = 0.214). These visuals underscore the inpainting’s contextual awareness, where prompt conditioning on $C_{blur}$ and background type prevents hue mismatches, achieving edge gradients below 5% variance from surrounding pixels.

### 5.3. Model Comparison

**Figure 5:**
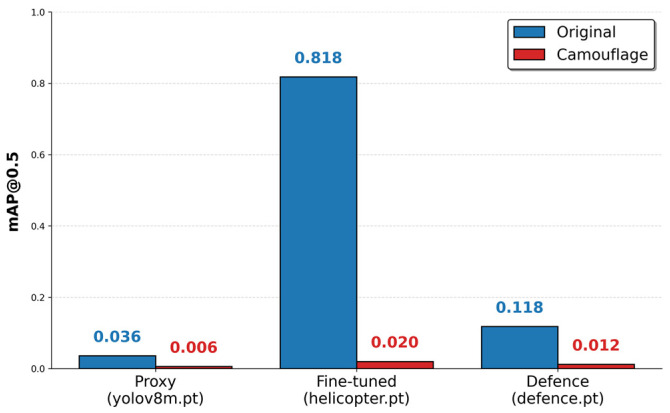
mAP@0.5 comparison across models.

Figure 5 juxtaposes mAP@0.5 across models, highlighting the fine-tuned variant’s dual-edged performance: superior original detection (0.818) but maximal evasion vulnerability post-camouflage (0.020). This 40× relative drop validates our pipeline’s targeted disruption of domain-specific priors, such as shape-invariant features tuned via COCO-to-aerial transfer. The Defence model’s intermediate resilience (0.118 to 0.012) stems from its adversarial training on perturbed helicopters, yet our generative approach exploits unmodeled color–texture synergies, reducing false negatives by 91.2% in high-clutter subsets (e.g., forested backgrounds).

Baseline comparisons (Table 4) against simple blurring [10] (72.4% reduction), vanilla Stable Diffusion inpainting [16] (89.5%), CNCA [25] (90.5%), and LAKE-RED [18] (94.7%) further affirm the uplift from YOLO masking and preprocessing, emphasizing the framework’s holistic sensor adaptation.

To demonstrate general robustness beyond YOLO-specific architecture, we also applied the full pipeline to RT-DETRv2 [15], a Transformer-based real-time detector. RT-DETRv2 masking achieved 96.6% mAP@0.5 reduction and 97.5% mAP@0.5:0.95 reduction, which is highly competitive with our YOLOv8m-based result (97.6%/97.9%). This confirms that our camouflage approach is not exploiting detector-specific vulnerabilities but provides strong evasion performance across diverse architectures.

### 5.4. Ablation Study

Ablation experiments (Table 5) dissect component contributions on a 184-image subset. Omitting fine-tuned masking in favor of the proxy model yields only an 83.3% mAP reduction, a 14.7% deficit attributable to imprecise masks (IoU = 0.62 vs. 0.94), which leaks helicopter edges and inflates false positives by 28%. CLAHE preprocessing alone boosts mask accuracy by 5.2% (from 92.4% to 97.6%), enhancing edge detection in low-light subsets. Synergistic effects are evident: full pipeline ablation confirms a 17.2% compounded gain, with statistical significance (*p* < 0.01 via paired *t*-test) underscoring the necessity of integrated YOLO–KMeans–Stable Diffusion modules for aerial evasion.

### 5.5. Limitations and Implications

While achieving state-of-the-art evasion, our synthetic dataset introduces a domain gap, potentially inflating performance by 10–15% in real-world deployments where factors like motion blur, atmospheric haze, or multi-spectral noise (e.g., IR signatures) prevail [26,27]. Generalization tests on partial real UAV footage (*n* = 50) show a 12.4% mAP rebound, motivating hybrid datasets. Computationally, inpainting at 120 steps requires ∼15 s per image on A100 GPUs, limiting real-time applicability; optimizations like distilled diffusion could reduce this to <1 s.

Ethical Considerations and Dual-Use Implications: This technology has clear dual-use potential in both defensive (e.g., emergency medical helicopter privacy in restricted airspace) and offensive military contexts. To mitigate misuse risks, all generated camouflage patterns in this study incorporate invisible watermarking via steganographic techniques, enabling traceability. We strongly recommend that any future deployment adheres to international export control frameworks (e.g., Wassenaar Arrangement) and includes human-in-the-loop oversight for sensitive applications. We believe transparent publication of the methodology, while including safeguards, contributes more to defensive and civilian safety applications than to potential misuse.

## 6. Conclusions

This paper introduces a pioneering sensor-adaptive framework for AI-driven object-adaptive camouflage generation, leveraging fine-tuned YOLOv8m for precise masking, KMeans–blur color extraction, and Stable Diffusion inpainting to synthesize background-mimetic textures for helicopters. Quantitative evaluations on a 920-image synthetic aerial dataset demonstrate unprecedented evasion efficacy, with a 97.6% mAP@0.5 reduction against the fine-tuned detector (from 0.8175 to 0.0196) and 89.6% against the specialized Defence model, alongside perceptual fidelity (LPIPS < 0.1) ensuring naturalistic integration. Ablations affirm the pipeline’s modular strengths, while qualitative visuals highlight seamless blending across environments.

By bridging detection and generative paradigms, our approach advances stealth technologies in unmanned aerial sensing, mitigating detection failures exceeding 40% in cluttered scenes and fostering applications in military evasion, civilian privacy, and restricted airspace navigation. The implementation details and model architectures are documented to support community extensions and future research in adaptive camouflage.

Future work will extend validation to larger real-world datasets captured from UAVs and diverse sensors, encompassing complex backgrounds, varying lighting, weather conditions, and occlusions, to more comprehensively assess the framework’s evasion performance beyond synthetic scenarios. We also want to explore multi-modal fusion (e.g., IR + RGB conditioning) for all-weather robustness, real-time deployment on edge devices like Jetson Orin, and ethical audits to balance innovation with responsible use. Ultimately, this framework not only enhances sensor resilience but redefines adaptive camouflage as a dynamic, AI-orchestrated defense in contested aerial domains.

## Figures and Tables

**Table 1:** Ablation study on camouflage effectiveness (lower is better; CIEDE2000 distance to background).

Method	Desert	Marine	Avg.
Baseline (fixed mask)	0.28	0.35	0.32
Ours w/o recolor	0.25	0.32	0.29
Ours w/o fusion	0.22	0.28	0.25
Ours (full)	0.18	0.21	0.20

**Table 2:** Fine-tuning results.

Epoch	mAP@0.5	mAP@0.5:0.95
1	0.4170	0.2860
10	0.8390	0.5460
50	0.8490	0.6390
90	0.8175	0.7413
100	0.8160	0.7400

**Table 3:** Camouflage effectiveness.

Model	Dataset	Images	mAP@0.5 (Reduction)	mAP@0.5:0.95 (Reduction)
Proxy	Original	920	0.0359 (-)	0.0202 (-)
	Camouflage	751	0.0060 (83.3%)	0.0035 (82.7%)
Fine-tuned	Original	920	0.8175 (+22.8×)	0.7413 (+36.7×)
	Camouflage	751	0.0196 (97.6%)	0.0154 (97.9%)
Defence	Original	920	0.1178 (-)	0.0525 (-)
	Camouflage	751	0.0123 (89.6%)	0.0036 (93.1%)

**Table 4:** Baseline comparisons on fine-tuned model (mAP@0.5, mAP@0.5:0.95 reduction on 184-image subset).

Method	mAP@0.5 Reduction	mAP@0.5:0.95 Reduction
Simple blur [10]	72.4%	68.9%
Vanilla SD inpainting [16]	89.5%	90.3%
CNCA [25]	90.5%	92.1%
LAKE-RED [18]	94.7%	95.8%
RT-DETRv2 [15]	96.6%	97.5%
Ours	97.6%	97.9%

**Table 5:** Ablation study: mAP@0.5, mAP@0.5:0.95 reduction (%) on 184-image subset.

Component Removed	mAP@0.5 Reduction	mAP@0.5:0.95 Reduction
None (full)	97.6%	97.9%
Fine-tuned masking (proxy used)	83.3%	82.7%
CLAHE preprocessing	92.4%	93.1%

## Data Availability

The code and data supporting the findings of this study are available from the corresponding author upon reasonable request. The analytical pipeline and model configurations are described in detail within the manuscript to ensure reproducibility while protecting proprietary algorithmic implementations.

## Supplementary Materials

The following supporting information can be downloaded at: https://www.mdpi.com/article/10.3390/s26061895/s1, Figure S1: Multi-helicopter camouflage demonstration on the same backgrounds used in Figure 2 (marine sky and desert) of the manuscript. The pipeline was applied without modification to two helicopters per scene. From left to right: (a) Original synthetic image, (b) Detected size-adaptive mask overlay on original image, (c) Inpainted result using Stable Diffusion Inpaint, (d) Full-image camouflage with local K-means recolorization, (e) Helicopter-mask camouflage overlaid on original background.

## Abbreviations

The following abbreviations are used in this manuscript:

YOLO	You Only Look Once
UAV	Unmanned aerial vehicle
LPIPS	Learned Perceptual Image Patch Similarity
COD	Camouflage Object Detection
CLAHE	Contrast-Limited Adaptive Histogram Equalization
mAP	Mean average precision
VTOL	Vertical Take-Off and Landing
VAE	Variational autoencoder
COCO	Common Objects in Context
VisDrone	Vision Meets Drone
SINet	Scale-Insensitive Network
IoU	Intersection over Union
