# Supplementary material for: AI-Driven Adaptive Camouflage Pattern Generation for Helicopter Detection Evasion in Aerial Sensor Imagery Using Fine-Tuned YOLOv8 and Stable Diffusion
Source: Sensors (Basel). 2026 Mar 17;26(6):1895. doi: 10.3390/s26061895 (PMC13029914; doi:10.3390/s26061895)
Supplement: Supplementary file 1 [file sensors-26-01895-s001.zip › sensors-4188423-supplementary.pdf]

Supplementary Materials

AI-Driven Adaptive Camouflage Pattern Generation for Helicopter Detection Evasion in Aerial Sensor Imagery Using Fine-Tuned YOLOv8 and Stable Diffusion

Jonghyeok Im, Yeonhong Kim, Heoung-Jae Chun and Kyoungsik Kim *

School of Mechanical Engineering, Yonsei University, 50 Yonsei-ro, Seodaemun-gu, Seoul 03722, Republic of Korea, kks@yonsei.ac.kr
* Correspondence: K.K.: kks@yonsei.ac.kr;

Section S1.

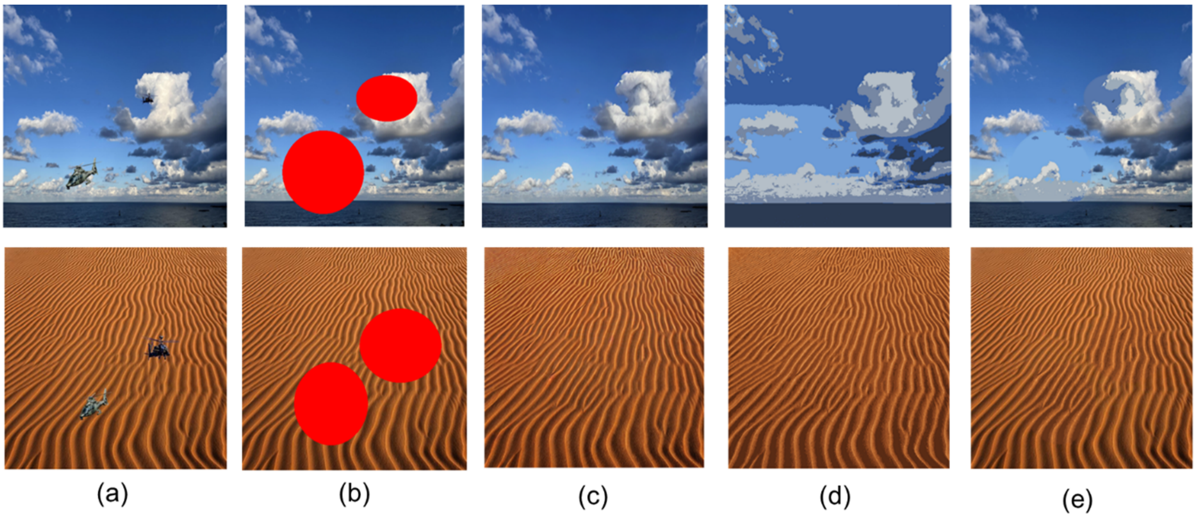

Figure S1. Multi-helicopter camouflage demonstration on the same backgrounds used in Figure 2 (marine sky and desert) of the manuscript. The pipeline was applied without modification to two helicopters per scene. From left to right: (a) Original synthetic image, (b) Detected size-adaptive mask overlay on original image, (c) Inpainted result using Stable Diffusion Inpaint, (d) Full-image camouflage with local K-means recolorization, (e) Helicopter-mask camouflage overlaid on original background.
